# Blockade of Arginine Vasopressin receptors prevents blood-brain barrier breakdown in Experimental Autoimmune Encephalomyelitis

**DOI:** 10.1038/s41598-019-57134-y

**Published:** 2020-01-16

**Authors:** Verónica Viñuela-Berni, Beatriz Gómez-González, Andrés Quintanar-Stephano

**Affiliations:** 1 Departamento de Fisiología y Farmacología. Centro de Ciencias Básicas, Universidad Autónoma de Aguascalientes, Aguascalientes, Aguascalientes, México; 2 Area of Neurosciences, Department of Biology of Reproduction, CBS, Universidad Autónoma Metropolitana, Unidad Iztapalapa, Mexico City, Mexico

**Keywords:** Neuroscience, Endocrinology

## Abstract

The blood-brain barrier (BBB) plays a significant pathophysiological role in multiple sclerosis (MS). Vasopressin (AVP) is released after brain injury and contributes to the inflammatory response. We propose that AVP may be modulating BBB permeability and hence affecting EAE clinical signs. Female Lewis rats were immunized s.c. with guinea-pig brain extract suspended in complete Freund’s adjuvant. Prior to that, animals were subjected to Neurointermediate pituitary lobectomy (NIL) or treated with AVP receptor antagonist (conivaptan). BBB permeability assays were performed. Western blot for claudin-5 and histological analysis were performed in conivaptan treated EAE rats. EAE increase in BBB permeability to Evans blue was reverted by the NIL surgery. AVP receptor blockade reverted the EAE BBB hyperpermeability to Evans blue and 10-kDa FITC-dextran in almost all brain regions. Both, AVP low levels and AVP receptor blockade attenuated EAE clinical signs. Conivaptan reduced perivascular cuffs in EAE rats. A decrease in claudin-5 expression was observed in EAE rats and conivaptan treatment partially restored normal levels. Our data indicate that V1a and V2 AVP receptors can modulate BBB permeability and consequently are involved in the CNS inflammatory process during EAE. Future research is required to characterize the utility of vasopressin antagonist in MS treatment.

## Introduction

The blood-brain barrier (BBB) is a dynamic barrier that maintains the homeostasis of the central nervous system (CNS). It is composed of a monolayer of brain endothelial cells and perivascular cells, including pericytes and astrocytes^1^. Brain endothelial cells are the main component of this barrier and form tight junctions that restrict the paracellular passage of molecules from blood-to-brain^2^. During neuroinflammation, leukocytes migrate and trigger inflammatory reactions in the CNS, causing BBB impairment. It has been widely described that the BBB plays an important pathophysiological role in neuroinflammatory diseases, such as multiple sclerosis (MS)^3–5^. MS is an autoimmune inflammatory demyelinating disease with different immune cells involved in its pathogenesis and T cells are the most recognized cell type^6^, and one of its most studied animal models is experimental autoimmune encephalomyelitis (EAE)^5^. CNS migration and accumulation of autoreactive myelin-specific T lymphocytes as well as BBB breakdown are the basic pathological hallmarks of both MS and EAE^7^. Th1 and Th17 cytokines are key factors modulating inflammatory responses and BBB impairment in both EAE and MS^8,9^.

Arginine vasopressin (AVP) plays an important pathophysiological role in various forms of brain injury, including cerebral ischemia, intracerebral hemorrhage, and traumatic cortical injury^10–14^. AVP-V1a receptors are broadly distributed in the rodent and human brain and spinal cord. Autoradiography as well as *in situ* hybridization studies have shown extensive AVP-V1a receptor distribution in cells of the cerebral cortex, hippocampus, pituitary gland and the choroid plexus^15,16^. Astrocytes, endothelial and perivascular smooth muscle cells present high mRNA AVP-V1a receptor expression^17,18^. AVP-V1b receptor expression is detected in kidneys, thymus, heart, lung, pancreas, adenohypophysis, spleen, and brain^19,20^.

It has been demonstrated that AVP is released as a response to peripheral inflammation and in brain injury AVP secretion also increases^21–25^. During brain injury AVP leads to cerebral vessel constriction and increased sympathetic tone, ensuing endothelial dysfunction and cerebral ischemia^26,27^. AVP also promotes dysregulation of ionic-water flux in the BBB through astrocytic Na + / K + ATPase, Na-K-Cl cotransporter (NKCC), and Aquaporin‐4 (AQP-4) dysfunction^11,28–30^, resulting in cytotoxic edema^30^. Brain ischemia can trigger vascular endothelial growth factor (VEGF) overexpression and increase endothelial permeability through AVP positive feedback^24^. On the other hand, the use of V1a and V2 receptor blockers, as conivaptan, in brain injury has been extensively reported; in fact, conivaptan reduces brain edema and BBB disruption during brain ischemia and cardiac arrest in mice^10,12^. All the above evidence suggests that AVP blockers can provide neuroprotection in conditions where BBB is compromised. In the present study, we investigated the effect of AVP receptor blockade with conivaptan on BBB permeability during EAE in rats.

## Results

### Experiment 1: AVP loss and AVP receptor blockade revert the BBB hyperpermeability to Evans blue in an EAE rat model

**Figure 1:**
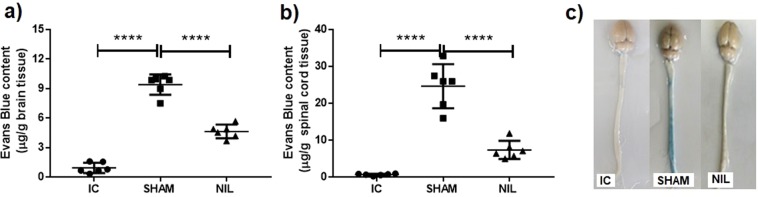
AVP deficiency by NIL surgery significantly attenuated EAE-induced blood brain barrier hyper-permeability to Evans blue. Six rats of every group were randomly assigned for the evaluation of BBB permeability using Evans blue extravasation assay. BBB permeability in NIL brain (**a**) and spinal cord (**b**) tissues was significantly decreased compared to the SHAM group. (**c**) Representative Evans blue extravasation in the brain and spinal cord of the intact control (IC), simulated operated animals immunized for Experimental Autoimmune Encephalomyelitis (SHAM) and animals with Neurointermediate Lobectomy of the pituitary plus EAE (NIL). Data represents mean ± SD, ****P < 0.0001, using a one-way ANOVA and Tukey´s post hoc test.

In order to investigate the possible mechanisms underlying the effects of AVP during EAE, the BBB permeability to Evans Blue was evaluated in the brain and spinal cord of Intact Control, SHAM-EAE and NIL-EAE animals. As shown in Fig. 1 EAE in SHAM group significantly increased BBB permeability to Evans blue in the brain (9.4 ± 1 µg/g wet tissue weight, Fig. 1a) and spinal cord (24.6 ± 5.9 μg/g wet tissue weight, Fig. 1b) as compared to the Intact Control (0.9 ± 0.5 and 0.6 ± 0.2 μg/g wet tissue weight respectively; p < 0.0001); and, interestingly, the NIL animals, characterized by AVP low levels, presented significant reduction in BBB permeability to Evans blue in the brain (4.65 ± 0.6 VS 9.4 ± 1 μg/g wet tissue weight, Fig. 1a) and in spinal cord (7.3 ± 0.6 vs 24.6 ± 5.9 μg/g wet tissue weight, Fig. 1b) as compared to the SHAM group (p < 0.0001). The amount of Evans blue tracer was higher in spinal cord tissue samples.

**Figure 2:**
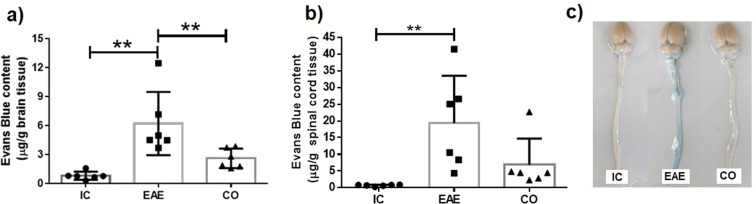
AVP V1a and V2 receptor antagonist reverts the EAE-induced blood-brain barrier hyper-permeability to Evans blue. Graphs show the concentration (µg/g of tissue) of Evans blue dye in the brain (**a**) and spinal cord (**b**) of Intact Control (IC). Experimental Autoimmune Encephalomyelitis (EAE) and EAE plus conivaptan at 3 mg/kg (CO) (n = 6 per group). (**a**) Conivaptan treatment significantly reduced Evans blue dye concentration in the brain as compared with the EAE group. (**b**) Graph shows a trend toward a decrease in Evans blue extravasation in animals treated with conivaptan, but no significant differences were found as compared with the EAE group. (**c**) Representative images of Evans blue extravasation in the brain and spinal cord of intact control (IC), Experimental Autoimmune Encephalomyelitis (EAE) and EAE plus conivaptan at 3 mg/kg (CO) groups. Mean ± SD, Kruskal-Wallis test and Dunn´s post hoc test, **p < 0.01.

To elucidate whether the blockade of AVP receptors has the same effect as AVP deficiency on BBB permeability to Evans blue, we used a synthetic antagonist of V1a and V2 AVP-receptors, conivaptan. As shown in Fig. 2, there was a significant increase in BBB permeability to Evans blue in EAE control group, both in brain (6.2 ± 3.2 VS 0.8 ± 0.4 μg/g wet tissue weight; p < 0.01, Fig. 2a) and spinal cord (19.38 ± 14.1 VS 0.6 ± 0.2 μg/g wet tissue weight; p < 0.01, Fig. 2b) as compared with the Intact Control. As expected, the BBB permeability to Evans blue in animals treated with conivaptan (CO; 3 mg/kg/day) was decreased both in brain (2.63 ± 1 VS 6.2 ± 3.2 μg/g wet tissue weight; p < 0.01) and spinal cord (6.9 ± 7.7 VS 19.38 ± 14.1 μg/g wet tissue weight; no significant differences), compared to the EAE group (Fig. 2a,b,c). This clearly indicates that AVP plays a pivotal role in regulating BBB physiology during a neuro-inflammatory process, like EAE. The Fig. 2c shows representative photographs of Evans blue staining in the spinal cord and brain for each group.

### Experiment 2: AVP receptor blockade decreases BBB hyperpermeability to 10 kDa FITC-dextrans in an EAE rat model

**Figure 3:**
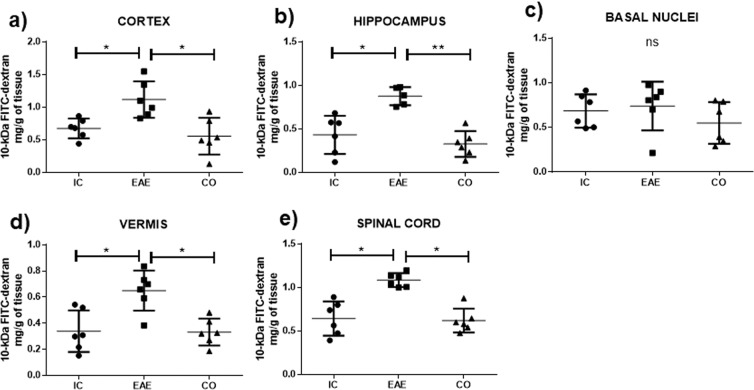
Conivaptan treatment reverts the EAE-induced blood-brain barrier hyper-permeability to 10-kDa FITC-dextran. Graphs show the concentration (mg/g of tissue) of 10-kDa FITC-dextran in the cortex (**a**), hippocampus (**b**), basal nuclei (**c**), vermis (**d**), and spinal cord (**e**) of intact control (IC), Experimental Autoinmmune Encephalomyelitis (EAE) and EAE plus conivaptan at 3 mg/kg (CO) (n = 6 per group). Mean ± SD. One-way ANOVA test and Tukey´s post hoc test, *p < 0.05 and **p < 0.01.

Due to the above results and the observations described previously on the regulatory effects of AVP on the BBB permeability, we decided to quantify BBB leakage to 10-kDa FITC-dextran tracer in specific CNS regions. Figure 3 shows that in the EAE group an increase in BBB permeability to 10 kDa FITC-dextran was observed in the cerebral cortex, hippocampus, cerebellar vermis, and spinal cord as compared to the intact control group. Again, conivaptan-treatment effectively suppressed EAE-induced BBB hyper-permeability to 10 kDa FITC-dextran in almost all brain regions. As shown in Fig. 3 the conivaptan-treated group presented a prominent decrease in the 10-kDa FITC-dextran concentration in the cortex (0.5 ± 0.2 mg/g wet tissue weight, p < 0.05), hippocampus (0.3 ± 0.1 mg/g wet tissue weight p < 0.01), cerebellar vermis (0.3 ± 0.1 mg/g wet tissue weight p < 0.05), and spinal cord (0.6 ± 0.1 mg/g wet tissue weight, p < 0.05) in comparison with the EAE group (cortex 1.2 ± 0.2 mg/g wet tissue weight, hippocampus 0.9 ± 0.1 mg/g wet tissue weight, vermis 0.7 ± 0.1 mg/g wet tissue weight, and spinal cord 1.1 ± 0.02 mg/g wet tissue weight). The 10 kDa FITC-dextran extravasation levels of EAE rats treated with conivaptan were indistinguishable from the intact controls. No significant differences were found in the basal nuclei (Fig. 3).

### Experiment 3: AVP receptor blockade partially reverts decreased tight junction protein expression in an EAE rat model

**Figure 4:**
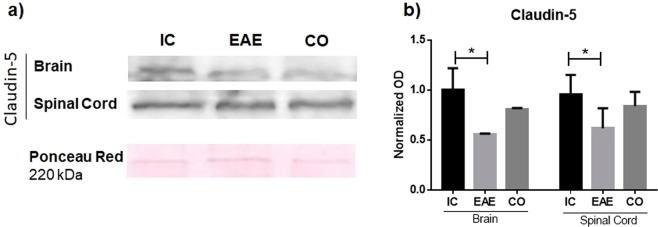
AVP receptor blockade partially reverts the decreased claudin-5 expression in the CNS of EAE rats. (**a**) Representative blot showing changes in the expression of the tight junction protein claudin‐5 in the brain and spinal cord of EAE group (EAE) and EAE conivaptan-treated group (CO) as compared with the intact controls (IC). (**b**) Graphs show the normalized optical density (OD) of protein expression for claudin‐5 in the IC, EAE and CO groups. A 220‐kDa band stained with Ponceau red was used for normalization of claudin-5 expression. Mean ± SD, *p ≤ 0.05 as compared with the intact control.

As increased BBB permeability is inversely related to tight junction assembly, we determined the expression levels of the tight junction protein claudin-5 by western blot in the EAE rats treated with conivaptan. As shown in Fig. 4 a large decrease in claudin-5 expression in the brain and spinal cord was observed in EAE rats (p < 0.05) (Fig. 4b). In the brain, the AVP receptor antagonist conivaptan partially reverted the EAE-induced low levels of claudin-5, the conivaptan-treated group did not differ significantly from both, the intact control and the EAE group.

### Experiment 4: AVP receptor blockade attenuates inflammatory cell infiltration and EAE neurological signs

**Figure 5:**
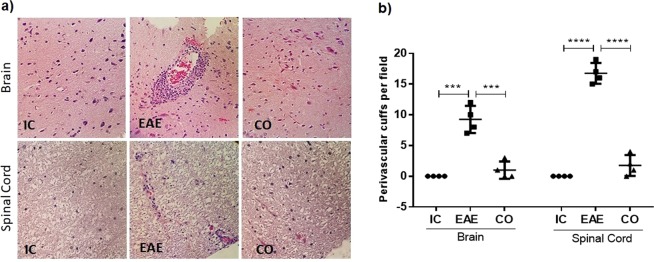
AVP receptor blockade attenuates inflammatory cell infiltration in the brain and spinal cord of rats with EAE. (**a**) Hematoxylin and eosin (H&E)-stained brain and spinal cord sections from intact control (IC), EAE, and conivaptan-treated EAE rats (CO). The EAE group shows perivascular cuffs in the brain and spinal cord, while EAE-CO (3 mg/kg) rats present a decrease in inflammatory cell infiltrates (H&E, X400). (**b**) Graph shows that perivascular cuffs decreased in both brain and spinal cord in the EAE CO-treated rats. Data are expressed as the mean ± SD. ***p < 0.001, ****p < 0.0001, Kruskal-Wallis test and Dunn´s post hoc test. There was no significant difference between intact control (IC) and conivaptan-treated (CO) groups in the number of perivascular cuffs.

Quantitative evaluation of hematoxylin and eosin stained sections showed the presence of infiltrated inflammatory cells (perivascular cuffs) in the brain (9.3 ± 2 counts) and spinal cord (16.8 ± 1.7 counts) of the EAE group (Fig. 5b). As it is shown in Fig. 5a,b the conivaptan-treated EAE group showed a prominet decrease in the number of perivascular cuffs both in the brain (1 ± 1.4 counts, p < 0.001) and the spinal cord (1.7 ± 1.7 counts, p < 0.0001) as compared to the EAE group.

**Figure 6:**
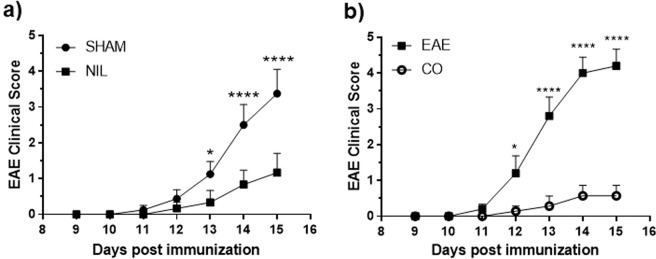
AVP deficiency and conivaptan treatment attenuate EAE clinical score. Graphs show the daily mean ± SEM of clinical scores from days 9 to 15 post-immunization among the EAE groups. (**a**) Significant differences between NIL (AVP deficient animals) and SHAM animals () were found from days 13 to 15 post-immunization. *p < 0.05, ****p < 0.0001, n = 10 animals per each experimental group. (**b**) Blockade of AVP V1a and V2 receptors ameliorates the disease time course of EAE. Significant differences between CO () and EAE () groups. Two-way ANOVA test, *p < 0.05, EAE vs CO at day 12 post-immunization. ****P < 0.0001 EAE vs CO at days 13, 14 and 15 post-immunization. n = 10 animals per each experimental group.

All animals were examined daily for neurological signs. SHAM operated and immunized rats (SHAM), used as a control to operated rats, developed EAE (90% incidence), with the onset of clinical signs occurring on days 10–11 after immunization. The SHAM operated and immunized rats reached its highest score on day 15, while in the NIL immunized group a significant decrease in the EAE clinical score was observed (0.3 ± 0.2 *versus* 2.25 ± 0.7 EAE clinical score at day 15 post immunization, p < 0.0001, Fig. 6a). Figure 6b shows that all groups of rats started to develop clinical signs at day 11 post EAE induction. Rats treated with vehicle developed a typical course of EAE, as evidenced by ascending paralysis beginning 11 days after immunization which peaked at day 15 post-immunization (mean score 4.2 and 90% of incidence). The treatment with 3 mg/kg/day conivaptan started 3 days pre-EAE induction and continued throughout the 2-week period post-EAE induction. Conivaptan treatment practically suppressed the EAE hind limb paralysis during the observation period (mean score 0.4; p < 0.0001, Fig. 6).

## Discussion

We showed that the loss of AVP by the NIL surgery induces a reduction in BBB permeability to Evans blue in the EAE rat model. These effects are in agreement with a significant reduction in severity of EAE clinical signs due to the AVP low levels induced by the NIL surgery. Moreover, here we show for the first time that conivaptan treatment effectively suppressed hindlimb paralysis during the 2-week follow up period of EAE development. In addition, conivaptan treatment restored normal BBB function in EAE rats. Under normal physiological conditions tracers as Evans blue and 10 kDa FITC-dextrans cross the BBB at very low levels. Our study showed that Evans blue and 10-kDa FITC-dextran extravasation was markedly increased in the SHAM and vehicle treated EAE rats compared with the control group. NIL surgery and conivaptan treatment significantly decreased BBB permeability compared with the SHAM and EAE group. Together, these results indicate that V1a and V2 AVP receptor antagonists could prevent the development of clinical signs and to restore the integrity of the disrupted BBB.

Our results are well in agreement with other experimental studies showing that AVP is involved in the pathophysiology of brain damage, Bhandari *et al*.^31^ effectively attenuated the brain edema secondary to intracerebral hemorrhage (ICH) in animals treated with Tolvaptan, an AVP-V2 receptor antagonist; they also showed a decrease in BBB permeability to Evans blue tracer in the Tolvaptan-treated ICH animals as compared to the ICH group^31^. Other animal experiments found that blockade of AVP-V1 receptors reduces brain edema and BBB disruption in stroke^32^. Clinical studies have shown that the plasma AVP level increases after ICH, stroke, and preeclampsia^23,32–35^.

The increased BBB permeability was related to a decreased expression of the tight junction protein claudin 5 in EAE and we showed in EAE conivaptan-treated rats an increase in brain and spinal cord expression of this tight junction protein. As claudin-5 is one of the most important tight junctions protein, its loss may actively contribute to severe BBB dysfunction.

AVP was reported to induce contraction responses in placental vessels^36^, Gao *et al*. showed that AVP acting on V1a receptor can induce vasoconstriction in placenta mainly via activation of the PKC pathway. The PKC family is known to affect endothelial barriers, and has been associated with alterations in tight junction protein assembly^37,38^, as well as in the integrity of the BBB in different inflammatory diseases^39^. In our model it may have occurred the same, the AVP antagonist blocked AVP receptor 1, which consequently translates into less PKC activation precluding tight junction alterations and BBB hyperpermeability in EAE rats. Also other authors have shown that AVP promotes the increased secretion of vascular endothelial growth factor (VEGF), triggering decreased expression of tight junction proteins and increased BBB permeability^24^. Furthermore, various studies have speculated that a relationship exists in the brain between AVP and AQP4^12,40^. An up-regulation of brain AQP4 was observed in many brain injury models and a reduction in AQP4 expression appeared after an AVP receptor blocker administration^41,42^. Likely, diverse experiments demonstrated that AVP in pregnancy is sufficient to up-regulate the expression of the co-stimulatory molecules CD80, CD86, and major histocompatibility complex class II (CLII) on dendritic cells^23^, which are necessary in the early pathogenesis of MS and EAE^7^ and induce a pro-inflammatory Th1 and Th17 profile, with elevated IFN-γ and IL-17^23^. These data indicate that conivaptan treatment may block all of those mechanisms during EAE, preventing BBB breakdown and the subsequent brain damage, ensuing the disappearance of clinical signs.

In MS or EAE an initiating factor is the loss of BBB function^3^, the BBB disruption is associated with changes in the basement membrane and tight junctions^43,44^. Elevated levels of IL-17A have been found in brain lesions and cerebrospinal fluid of MS patients^45,46^ and IL-17A facilitated migration of human Th17 cells through the BBB by disrupting endothelial cell tight junctions^47^. Matrix metalloproteinases (MMPs) and TNF-α have also been implicated in the breakdown of the BBB in MS and EAE^48^. In the most common form of MS, relapse-remitting (RR) MS, the lesions are centered on blood vessels, which become inflamed and leaky, ensuing edema formation. Other works provided evidence that a luminal BBB membrane Na–K–Cl cotransporter is a major contributor to edema formation^28^, and studies using cultured cerebral endothelial cells found that the cotransporter is quite sensitive to AVP stimulation^29^.

We have previously shown that AVP-deficient rats (Neuro-Intermediate pituitary Lobectomy) decreased incidence and severity of EAE, and that effect coincided with a significant reduction in spinal cord perivascular cuffs^49^. In the present study we show that conivaptan treatment also significantly decreased the number of perivascular cuffs in brain and spinal cord. This observation is consistent with our present’s results which showed a partial restoration of claudin-5 expression and decreased severity of the neurological signs in conivaptan treated EAE rats.

Evidence is accumulating that AVP plays a major role in the regulation of neuroinflammatory reactions^10,41^. Our data reveal that inhibition of V1a and V2 AVP receptors can prevent BBB hyper-permeability caused by EAE. The present study provides a new insight into how AVP may regulate BBB exchange during inflammatory response in the EAE rat model. The underlying pathophysiological mechanism and possible clinical importance of the present findings deserves further investigation.

In conclusion, we found that the blockade of the AVP V1a and V2 receptors with conivaptan could protect the BBB, contributing to the amelioration of neurological disorder in an EAE rat model. Conivaptan may be a promising prophylactic candidate to control neurological disease (MS) severity by targeting the BBB.

## Methods

Female Lewis rats 10–12 weeks old were used and were obtained from the Autonomous University of Aguascalientes. The animal´s room was kept under controlled temperature (22–24 °C) and light/dark conditions (12 h light/dark cycle, lights on at 7am). The diet consisted of Purina rat chow and tap water *ad libitum*. The animals were divided into five groups: Intact Control (IC group), SHAM group, immunized for EAE (EAE group), Neurointermediate pituitary lobectomized rats and immunized for EAE (NIL group) and immunized for EAE treated with Conivaptan (CO group). The animals were habituated for 7 days to the room conditions prior to the experiment. All surgical experimental procedures were approved by the Institutional Animal Care and Use Committee of the Universidad Autónoma de Aguascalientes and are compatible with the Institute for Laboratory Animal Research (USA, 1996) guidelines.

### Neurointermediate pituitary lobectomy (NIL) surgery

NIL surgeries were performed under anesthesia with mixture of O2/Sevorane, the neck was shaved, and rats were placed in dorsal decubitus position. The intermediate and posterior lobes (neurointermediate lobe) of the pituitary gland were removed under a dissecting microscope through the parapharyngeal-transoccipital-sphenoidal approach and gentle aspiration via a bent needle. The method employed has been described previously^49,50^. Only successfully operated NIL rats with complete removal of the neurointermediate lobe and without damage to the anterior lobe were included in the study. Removal of neurointermediate lobe o the pituitary generated AVP low levels as previously reported^51^. As a control condition, in the SHAM group the pituitary gland was only exposed but not removed. All animals were injected with penicillin (5000 IU, i.m.) once a day for 7 days and metamizole (15 mg/kg; i.m.) once daily for 3 days.

### EAE immunization

Rats were anesthetized with inhalatory anesthesia (Sevorane), the tail base was shaved, and rats were placed in the ventral decubitus position. Animals were injected subcutaneously at the dorsal base of the tail with 200 µl of an emulsion composed of 50% guinea pig brain homogenate emulsified with complete Freund’s adjuvant. The emulsion was composed of 1 ml brain homogenate, 1 ml incomplete Freund’s adjuvant (F5506, Sigma, St. Louis, Mo., USA) and 2 mg of finely ground Mycobacterium tuberculosis (strain H37 Ra, Difco, Detroit, Mich., USA).

### Drug treatment

Dimethyl sulfoxide (DMSO) (EAE; 10% i.m.) or conivaptan (CO; 3 mg/kg of body weight i.m.) (ShangHai Biochempartner Co., Ltd China) were administrated to EAE and CO groups respectively. Administration started 3 days before EAE immunization and continued daily until the end of the experiment on day 15 after immunization (AI).

### Evaluation of EAE

Rats were examined daily for disease signs and were assigned scores on a conventional scale^52,53^ as following: 0, healthy; 1, tail paralysis; 2, ataxia in one hind limb; 3, paralysis of one hind limb; 4, paralysis of one hind limb and ataxia of the other, and 5, complete paralysis of tail and both hind limbs. The intact control group was also evaluated as negative control.

### Experiment 1: BBB permeability assay to evans blue

The integrity of the BBB was determined using Evans blue (EB) as a tracer^54^. Rats were divided into intact control (IC, n = 6), simulated operated rats plus EAE (SHAM, n = 6) and Neurointermediate pituitary lobectomized rats plus EAE (NIL = 6). An independent group of rats was divided into intact control (IC, n = 6), Experimental Autoimmune encephalomyelitis animals (EAE, n = 6) and treated animals with 3 mg/kg of conivaptan plus EAE (CO, n = 6). On day 15 after immunization rats were anesthetized with a mixture of O2/Sevorane and then injected with 2% Evans blue dye in PBS at a dose of 2 mL/kg through the jugular vein. Two hours later, animals were anesthetized with pentobarbital (ip. 0.063 g/kg body weight) and then sacrificed. Immediately after rats were transcardially perfused with PBS (200 ml) at a rate of 20 ml/min. The brain and spinal cord were collected, and their wet weights determined. The organ samples were homogenized in 3 ml N, N-dimethylformamide^55^. After 24 h of incubation at 55 °C, the organ samples were centrifuged at 14,000 rpm for 20 min at 20 °C. Supernatant was collected and absorbance was measured at 620 nm using a Jenaway 6300 spectrophotometer. The quantity of extravasated dye was calculated by measuring the content of Evans blue in the brain and spinal cord tissues from a linear standard curve derived from known amounts of the dye and was expressed as micrograms per gram of each tissue.

### Experiment 2: Blood-brain barrier permeability assay to 10-kDa FITC-dextrans

10-kDa FITC-dextrans (Sigma FD10S) were used to evaluate blood–brain barrier permeability to low-molecular weight tracers. Dextrans were suspended in phosphate buffered saline (PBS) to obtain a concentration of 3 mg/ml. Rats were divided into intact control group (IC, n = 6), Experimental Autoimmune encephalomyelitis group (EAE, n = 6) and EAE plus 3 mg/kg of conivaptan (CO, n = 6). Rats were anesthetized with sodium pentobarbital (ip. 0.063 g/ kg body weight), and 0.2 mL/100 g body weight of FITC-dextran was administrated in the jugular vein. After 10 minutes of FITC-dextran circulation, rats were perfused during 5 minutes with PBS at a rate of 20 ml/min. The brain and spinal cord were removed; the concentration of 10-kDa FITC-dextran was calculated in the hippocampus, basal nuclei, cerebellar vermis, cerebral cortex, and spinal cord. Samples were weighed, homogenized with PBS and centrifuged at 14,000 rpm for 10 min at 4 °C. FITC-Dextran fluorescence was quantified using fluorescence microplate reader at 520 nm (SpectraMax M5, Molecular Device)^56^. Fluorescence values from duplicate wells were fit to a standard curve built from known amounts of the tracer. Results are showed as the concentration of FITC-dextran per weight of each tissue (mg/g).

### Experiment 3: Western blot of tight junction protein claudin-5

Animals from the intact control group (IC, n = 3), Experimental Autoimmune encephalomyelitis group (EAE, n = 3) and EAE plus 3 mg/kg of conivaptan (CO, n = 3) were euthanized between 08:00 hours and 10:00 hours. Deaths occurred at the 15th day post-inmunization. Microvessels were isolated from brain and spinal cord. Briefly, tissues were placed in cold sucrose buffer (sucrose 10.95%, Hepes 0.07% and BSA 1%, pH 7.4, SB), and homogenized. After two centrifugation rounds (3500 rpm 10 min each) at 4 °C, the supernatant was discarded, and the pellet was resuspended in cold bovine serum. Samples were then centrifuged at 1130 rpm for 50 seconds at 4 °C three times and the supernatants obtained. The supernatants were centrifuged again at 7000 g for 5 min at 4 °C. The supernatant was discarded, and the pellet washed in PBS, then the pellet was resuspended in RIPA buffer with protease inhibitors. The samples were centrifuged at 13,500 rpm for 10 min at 4 °C, the supernatant was collected, and protein concentration determined by the Bradford assay (500‐0006, BioRad). The proteins (30 μg) were resolved using a denaturing 10% sodium dodecyl sulphate–polyacrylamide gel electrophoresis (SDS–PAGE) and then transferred to PVDF membranes. Membranes were blocked with low‐fat milk, followed by incubation with the primary antibody anti‐claudin 5 (1:1,000; Thermo Fisher) overnight at 4 °C. After two washes, the membranes were incubated with biotin‐secondary antibody followed by avidin‐biotin complex (ABC kit, Vector Labs PK6100), and were revealed with a chemiluminescence detection system (Inmobilion Western WBKLS0500). Blot images were acquired using the C‐Digit equipment and the Li‐COR image studio (Version 3.1). A 220 kDa band stained with Ponceau red was used for normalization. Experiments were performed by duplicate.

### Experiment 4: Histological analysis of perivascular cuffs in the CNS

Rats were divided into intact control group (IC, n = 4), Experimental Autoimmune encephalomyelitis group (EAE, n = 4) and EAE plus 3 mg/kg of conivaptan (CO, n = 4). At 15 days post-immunization animals were anesthetized with sodium pentobarbital (ip. 0.063 g/kg body weight) and then sacrificed, the brain and spinal cord were dissected and fixed in 10% neutral buffered formalin, embedded in paraffin and sectioned into 6 μm slides. The sections were stained with hematoxylin and eosin to evaluate inflammatory cell infiltration. One photograph *per* slide was obtained for each subject with a magnification of x400. The inflammatory perivascular cuffs were counted on the entire area at each section level and photographed using a Nikon light microscope (Optiphot-2) using a CCD.

### Statistical analysis

Statistical significance was analyzed by one-way ANOVA and Kruskal-Wallis test for permeability assays, western blot analysis and histological analysis. A two-way ANOVA tests was used for statistical analysis of EAE clinical signs. Differences were considered significant at p < 0.05. The results are presented as mean ± SD. Statistical analysis was performed with the GraphPad Prism 7 (GraphPad Software, San Diego, CA, USA).

## Supplementary Material

Supplementary information.

## References

[1] Stamatovic SM, Johnson AM, Keep RF, Andjelkovic AV (2016). Junctional proteins of the blood-brain barrier: new insights into function and dysfunction. Tissue barriers.

[2] Keaney J, Campbell M (2015). The dynamic blood–brain barrier. The FEBS journal.

[3] Bennett J (2010). Blood–brain barrier disruption and enhanced vascular permeability in the multiple sclerosis model EAE. Journal of neuroimmunology.

[4] V Borlongan C, A Rodrigues A, Carolina Oliveira M (2012). Breaking the barrier in stroke: what should we know? A mini-review. Current pharmaceutical design.

[5] Lee MJ (2016). IKKβ-mediated inflammatory myeloid cell activation exacerbates experimental autoimmune encephalomyelitis by potentiating Th1/Th17 cell activation and compromising blood brain barrier. Molecular neurodegeneration.

[6] Høglund RA, Maghazachi AA (2014). Multiple sclerosis and the role of immune cells. World journal of experimental medicine.

[7] Hemmer B, Kerschensteiner M, Korn T (2015). Role of the innate and adaptive immune responses in the course of multiple sclerosis. The Lancet Neurology.

[8] Procaccini C, De Rosa V, Pucino V, Formisano L, Matarese G (2015). Animal models of multiple sclerosis. European journal of pharmacology.

[9] Gao Q (2016). Blockade of CD47 ameliorates autoimmune inflammation in CNS by suppressing IL-1-triggered infiltration of pathogenic Th17 cells. Journal of autoimmunity.

[10] Zeynalov E, Jones SM, Seo J-W, Snell LD, Elliott JP (2015). Arginine-vasopressin receptor blocker conivaptan reduces brain edema and blood-brain barrier disruption after experimental stroke in mice. PloS one.

[11] Ameli PA (2014). Role of vasopressin and its antagonism in stroke related edema. Journal of neuroscience research.

[12] Liu X, Nakayama S, Amiry-Moghaddam M, Ottersen OP, Bhardwaj A (2010). Arginine-vasopressin V 1 but not V 2 receptor antagonism modulates infarct volume, brain water content, and aquaporin-4 expression following experimental stroke. Neurocritical care.

[13] Szmydynger-Chodobska J, Gandy JR, Varone A, Shan R, Chodobski A (2013). Synergistic interactions between cytokines and AVP at the blood-CSF barrier result in increased chemokine production and augmented influx of leukocytes after brain injury. PLoS One.

[14] Hedna VS (2014). Treatment of stroke related refractory brain edema using mixed vasopressin antagonism: a case report and review of the literature. BMC neurology.

[15] Campbell P, Ophir AG, Phelps SM (2009). Central vasopressin and oxytocin receptor distributions in two species of singing mice. Journal of Comparative Neurology.

[16] Ostrowski N, Lolait S, Young W (1994). Cellular localization of vasopressin V1a receptor messenger ribonucleic acid in adult male rat brain, pineal, and brain vasculature. Endocrinology.

[17] Szmydynger-Chodobska J (2004). Increased expression of vasopressin v1a receptors after traumatic brain injury. Journal of neurotrauma.

[18] Kozniewska E, Romaniuk K (2008). Vasopressin in vascular regulation and water homeostasis in the brain. J Physiol Pharmacol.

[19] Saito M, Sugimoto T, Tahara A, Kawashima H (1995). Molecular cloning and characterization of rat V1b vasopressin receptor: evidence for its expression in extrapituitary tissues. Biochemical and biophysical research communications.

[20] Hernando F, Schoots O, Lolait SJ, Burbach JPH (2001). Immunohistochemical localization of the vasopressin V1b receptor in the rat brain and pituitary gland: anatomical support for its involvement in the central effects of vasopressin. Endocrinology.

[21] Liu X (1992). An experimental study of arginine vasopressin on acute ischemic brain edema in gerbils (1). Zhonghua shen jing jing shen ke za zhi= Chinese journal of neurology and psychiatry.

[22] Barreca T (2001). Evaluation of the secretory pattern of plasma arginine vasopressin in stroke patients. Cerebrovascular Diseases.

[23] Scroggins SM (2018). Elevated vasopressin in pregnant mice induces T-helper subset alterations consistent with human preeclampsia. Clinical Science.

[24] Largeau, B. *et al*. Arginine Vasopressin and Posterior Reversible Encephalopathy Syndrome Pathophysiology: the Missing Link? *Molecular Neurobiology*, 1–15 (2019). 10.1007/s12035-019-1553-y 30924075

[25] Cintra EdA (2004). Vasopressin serum levels in patients with severe brain lesions and in brain-dead patients. Arquivos de neuro-psiquiatria.

[26] Fernández N, Martínez MA, García‐Villalón AL, Monge L, Diéguez G (2001). Cerebral vasoconstriction produced by vasopressin in conscious goats: role of vasopressin V1 and V2 receptors and nitric oxide. British journal of pharmacology.

[27] Nakai M (1987). Contractile effects of perivascularly applied vasopressin on the pial artery of the cat brain. The Journal of physiology.

[28] O’donnell ME (2013). Intravenous HOE-642 reduces brain edema and Na uptake in the rat permanent middle cerebral artery occlusion model of stroke: evidence for participation of the blood–brain barrier Na/H exchanger. Journal of Cerebral Blood Flow & Metabolism.

[29] Hertz L, Xu J, Chen Y, E Gibbs M, Du T (2014). Antagonists of the Vasopressin V1 Receptor and of the β1-Adrenoceptor Inhibit Cytotoxic Brain Edema in Stroke by Effects on Astrocytes-but the Mechanisms Differ. Current neuropharmacology.

[30] Wolburg-Buchholz K (2009). Loss of astrocyte polarity marks blood–brain barrier impairment during experimental autoimmune encephalomyelitis. Acta neuropathologica.

[31] Bhandari S (2017). A systematic review of known interventions for the treatment of chronic nonhypovolaemic hypotonic hyponatraemia and a meta-analysis of the vaptans. Clinical endocrinology.

[32] Vakili A, Kataoka H, Plesnila N (2005). Role of arginine vasopressin V1 and V2 receptors for brain damage after transient focal cerebral ischemia. Journal of Cerebral Blood Flow & Metabolism.

[33] Simard M, Nedergaard M (2004). The neurobiology of glia in the context of water and ion homeostasis. Neuroscience.

[34] Trabold R, Krieg S, Schöller K, Plesnila N (2008). Role of vasopressin V1a and V2 receptors for the development of secondary brain damage after traumatic brain injury in mice. Journal of neurotrauma.

[35] Santillan MK (2014). Vasopressin in preeclampsia: a novel very early human pregnancy biomarker and clinically relevant mouse model. Hypertension.

[36] Gao, Q. *et al*. Hyper-methylation of AVPR1A and PKCΒ gene associated with insensitivity to arginine vasopressin in human pre-eclamptic placental vasculature. *EBioMedicine* (2019). 10.1016/j.ebiom.2019.05.056 PMC6606951 31175056

[37] Willis CL, Meske DS, Davis TP (2010). Protein kinase C activation modulates reversible increase in cortical blood–brain barrier permeability and tight junction protein expression during hypoxia and posthypoxic reoxygenation. Journal of Cerebral Blood Flow & Metabolism.

[38] Andreeva AY, Krause E, Müller E-C, Blasig IE, Utepbergenov DI (2001). Protein kinase C regulates the phosphorylation and cellular localization of occludin. Journal of Biological Chemistry.

[39] Fleegal MA, Hom S, Borg LK, Davis TP (2005). Activation of PKC modulates blood-brain barrier endothelial cell permeability changes induced by hypoxia and posthypoxic reoxygenation. American Journal of Physiology-Heart and Circulatory Physiology.

[40] Jia S-W, Liu X-Y, Wang SC, Wang Y-F (2016). Vasopressin hypersecretion-associated brain edema formation in ischemic stroke: underlying mechanisms. Journal of Stroke and Cerebrovascular Diseases.

[41] Manaenko A (2011). Arginine-vasopressin V1a receptor inhibition improves neurologic outcomes following an intracerebral hemorrhagic brain injury. Neurochemistry international.

[42] Niermann H, Amiry-Moghaddam M, Holthoff K, Witte OW, Ottersen OP (2001). A novel role of vasopressin in the brain: modulation of activity-dependent water flux in the neocortex. Journal of Neuroscience.

[43] Tenreiro M, Ferreira R, Bernardino L, Brito M (2016). Cellular response of the blood-brain barrier to injury: potential biomarkers and therapeutic targets for brain regeneration. Neurobiology of Disease.

[44] Luissint A-C, Artus C, Glacial F, Ganeshamoorthy K, Couraud P-O (2012). Tight junctions at the blood brain barrier: physiological architecture and disease-associated dysregulation. Fluids and Barriers of the CNS.

[45] Lock C (2002). Gene-microarray analysis of multiple sclerosis lesions yields new targets validated in autoimmune encephalomyelitis. Nature medicine.

[46] Matusevicius D (1999). Interleukin-17 mRNA expression in blood and CSF mononuclear cells is augmented in multiple sclerosis. Multiple Sclerosis Journal.

[47] Yamamura, T. & Gran, B. *Multiple Sclerosis Immunology: A Foundation for Current and Future Treatments*. (Springer Science & Business Media, 2013).

[48] Kirk J, Plumb J, Mirakhur M, McQuaid S (2003). Tight junctional abnormality in multiple sclerosis white matter affects all calibres of vessel and is associated with blood–brain barrier leakage and active demyelination. The Journal of Pathology: A Journal of the Pathological Society of Great Britain and Ireland.

[49] Quintanar-Stephano A, Organista-Esparza A, Chavira-Ramírez R, Kovacs K, Berczi I (2012). Effects of neurointermediate pituitary lobectomy and desmopressin on acute experimental autoimmune encephalomyelitis in lewis rats. Neuroimmunomodulation.

[50] BEN-JONATHAN N, PETERS LL (1982). Posterior pituitary lobectomy: differential elevation of plasma prolactin and luteinizing hormone in estrous and lactating rats. Endocrinology.

[51] Villanueva-Rodriguez GM (2016). Effects of arginine vasopressin (AVP) deficiency on long term arterial blood pressure in normal and spontaneously hypertensive rats (SHR). The FASEB Journal.

[52] Becher B, Durell BG, Miga AV, Hickey WF, Noelle RJ (2001). The clinical course of experimental autoimmune encephalomyelitis and inflammation is controlled by the expression of CD40 within the central nervous system. Journal of Experimental Medicine.

[53] Quintanar-Stephano A, Chavira-Ramírez R, Kovacs K, Berczi I (2005). Neurointermediate pituitary lobectomy decreases the incidence and severity of experimental autoimmune encephalomyelitis in Lewis rats. Journal of endocrinology.

[54] Dal-Pizzol F (2013). Matrix metalloproteinase-2 and metalloproteinase-9 activities are associated with blood–brain barrier dysfunction in an animal model of severe sepsis. Molecular neurobiology.

[55] Wang X-S (2014). Idazoxan reduces blood–brain barrier damage during experimental autoimmune encephalomyelitis in mouse. European journal of pharmacology.

[56] Hurtado-Alvarado G, Domínguez-Salazar E, Velázquez-Moctezuma J, Gómez-González B (2016). A2A adenosine receptor antagonism reverts the blood-brain barrier dysfunction induced by sleep restriction. PloS one.

