# Supplementary material for: Blockade of Arginine Vasopressin receptors prevents blood-brain barrier breakdown in Experimental Autoimmune Encephalomyelitis
Source: Sci Rep. 2020 Jan 16;10:467. doi: 10.1038/s41598-019-57134-y (PMC6965180; doi:10.1038/s41598-019-57134-y)
Supplement: Supplementary file 1 — Supplementary information. [file 41598_2019_57134_MOESM1_ESM.pdf]

Blockade of Arginine Vasopressin receptors prevents blood-brain barrier breakdown in Experimental Autoimmune Encephalomyelitis

Authors:

Verónica Viñuela-Berni[1], Beatriz Gómez-González[2], and Andrés Quintanar-Stephano[1]*

[1]Departamento de Fisiología y Farmacología. Centro de Ciencias Básicas, Universidad Autónoma de Aguascalientes, Aguascalientes, Aguascalientes. México.

[2] Area of Neurosciences, Department of Biology of Reproduction, CBS, Universidad Autónoma Metropolitana, Unidad Iztapalapa, Mexico City, Mexico

Supplementary figure 1

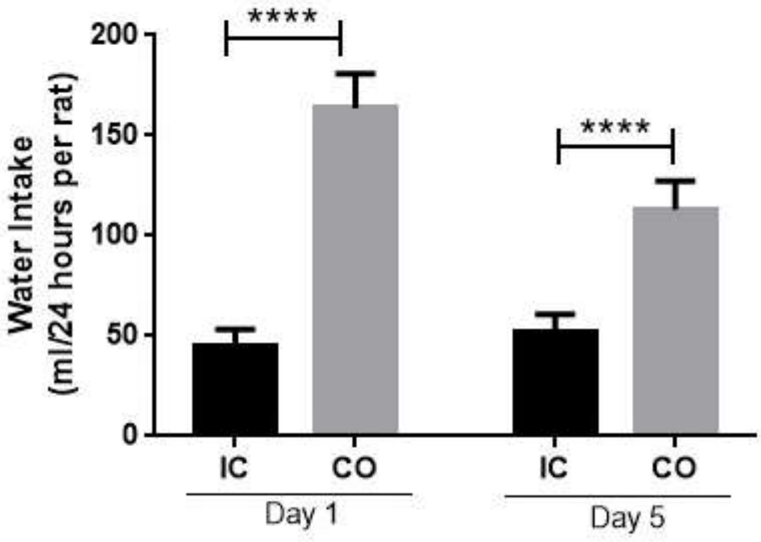

Supplementary Figure 1. Effects of conivaptan treatment on water intake. Graphs show the milliliters per rat in the first and fith day of conivaptan administration 3 mg/kg (CO) (n = 10 per group). Mean ± SD, one-tail t-test was used, p****<0.0001.
